# In-and-Out Molecular Changes Linked to the Type 2 Diabetes Remission after Bariatric Surgery: An Influence of Gut Microbes on Mitochondria Metabolism

**DOI:** 10.3390/ijms19123744

**Published:** 2018-11-24

**Authors:** Paulina Samczuk, Hady Razak Hady, Edyta Adamska-Patruno, Anna Citko, Jacek Dadan, Coral Barbas, Adam Kretowski, Michal Ciborowski

**Affiliations:** 1 Clinical Research Centre, Medical University of Bialystok, 15-276 Bialystok, Poland; edyta.adamska@umb.edu.pl (E.A.-P.); ankacitko@gmail.com (A.C.); adamkretowski@wp.pl (A.K.); michal.ciborowski@umb.edu.pl (M.C.); 2 1st Clinical Department of General and Endocrine Surgery, Medical University of Bialystok, 15-276 Bialystok, Poland; hadyrazakh@wp.pl (H.R.H.); klchirog@umb.edu.pl (J.D.); 3 Center for Metabolomics and Bioanalysis (CEMBIO), Universidad CEU-San Pablo, km 0, Urbanización Montepríncipe. M-501, Alcorcón, 28925 Madrid, Spain; cbarbas@ceu.es; 4 Department of Endocrinology, Diabetology and Internal Medicine, Medical University of Bialystok, 15-276 Bialystok, Poland

**Keywords:** bariatric surgery, metabolomics, diabetes remission, laparoscopic sleeve gastrectomy, LC-MS, GC-MS

## Abstract

Different kinds of gastrointestinal tract modulations known as “bariatric surgery” are actually the most effective treatment for obesity and associated co-morbidities, such as type 2 diabetes (T2DM). The potential causes of those effects have yet to be explained. In our study, we focused on molecular changes evoked by laparoscopic sleeve gastrectomy leading to T2DM remission. Two complementary metabolomics techniques, namely, liquid chromatography coupled with mass spectrometry (LC-MS) and gas chromatography mass spectrometry (GC-MS), were used to study those effects in a group of 20 obese patients with T2DM selected from a cohort of 372 obese individuals who underwent bariatric surgery and did not receive anti-diabetic treatment afterward. Modified levels of carnitines, lipids, amino acids (including BCAA) and α- and β-hydroxybutyric acids were detected. Presented alterations suggest a major role of mitochondria activity in T2DM remission process. Moreover, some of the observed metabolites suggest that changes in gut microbiota composition may also correlate with the tempo of diabetes recovery. Additional analyses confirmed a relationship between biochemical and clinical parameters and the aforementioned metabolites, thereby, highlighting a role of mitochondria and microbes. Our data suggests that there is a previously undescribed relationship between mitochondria and gut microbiota, which changes after the bariatric surgery. More investigations are needed to confirm and explore the observed findings.

## 1. Introduction

Type 2 diabetes (T2DM) remission after bariatric surgery is a fact. Thus, gastrointestinal metabolic surgery is actually the most effective treatment for obesity and associated co-morbidities, like T2DM [1,2,3].

Bariatric surgery refers to three parts of anatomical gastrointestinal tract modulations-gastric restriction, exclusion of duodenum and upper intestine, and rapid delivery of food to the distal intestine or short common channel. These anatomical changes may induce a lot of physiologic and molecular changes helping to resolve type 2 diabetes [2]. The proposed mechanisms underlying diabetes remission after metabolic surgery included the starvation-followed-by weight-loss hypothesis, the ghrelin hypothesis, the lower intestinal (hind-gut) hypothesis and the upper intestinal (fore-gut) hypothesis. More theories have been proposed recently, including changes in bile acid metabolism and intestinal microbiome. Interestingly, none of these theories necessarily negate the others. These modulations result in reduced hepatic glucose, increased tissue glucose uptake, improved insulin sensitivity and enhanced β-cell function [1,2,4,5].

Additionally, a discussion remains on which factors can play an essential role in predicting the impact of bariatric surgery on T2DM. Age, BMI, C-peptide, duration of the disease (ABCD score), HbA1c, fasting blood glucose, incretins glucagon-like peptide-1 (GLP-1) and gastric inhibitory peptide (GIP)] can be included. Another example of a score grading system that is used to predict T2DM remission after bariatric surgery is the DiaRem score. It includes factors such as age, HbA1c, antidiabetic drugs and insulin, which are used to predict the remission T2DM. However, the DiaRem also has some limitations, e.g., the duration of T2DM omission [6]. Therefore, T2DM remission is probably an effect of a combination of a few mechanisms [7] and it can be difficult to design a study to elucidate a single mechanism. Fortunately, nowadays, we own research tools that allow the following of even the smallest modifications of numerous molecular factors in a single analysis, i.e., the “omics” sciences—genomics, transcriptomics, proteomics and metabolomics.

The potential of metabolomics in clinical research is well established. This includes studies related to diabetes, obesity and different aspects of bariatric procedures. Roux-en-Y gastric bypass (RYGB) and sleeve gastrectomy are the most frequently examined procedures; these have been routinely studied using metabolomics platforms such as liquid chromatography coupled with mass spectrometry (LC-MS) and/or hydrogen nuclear magnetic resonance (^1^H-NMR). Currently, most of the studies are focused on human or mice/rats samples with serum or plasma as the most commonly used biological materials. Although the influence of bariatric surgery on morbid obesity and type 2 diabetes were investigated the most frequently, other obesity-related diseases and health conditions were also occasionally studied [8].

While laparoscopic sleeve gastrectomy (LSG) along with RYGB are nowadays considered the “gold standard” of bariatric procedures [8,9], sleeve gastrectomy has become one of the most commonly used primary bariatric procedures for morbid obesity [10]. Moreover, LSG leads to significant improvement in biochemical glucose homeostasis and may be considered as a method for T2DM treatment [10]. Although molecular background of mechanisms underlying type 2 diabetes remission after bariatric surgery has been heavily investigated, it remains intriguing and has yet to be well defined [2,8,11]. Consequently, the aim of this study was to apply two complementary metabolomics platforms to investigate molecular changes accompanying early T2DM remission after LSG procedure. 

## 2. Results

**Figure 1:**
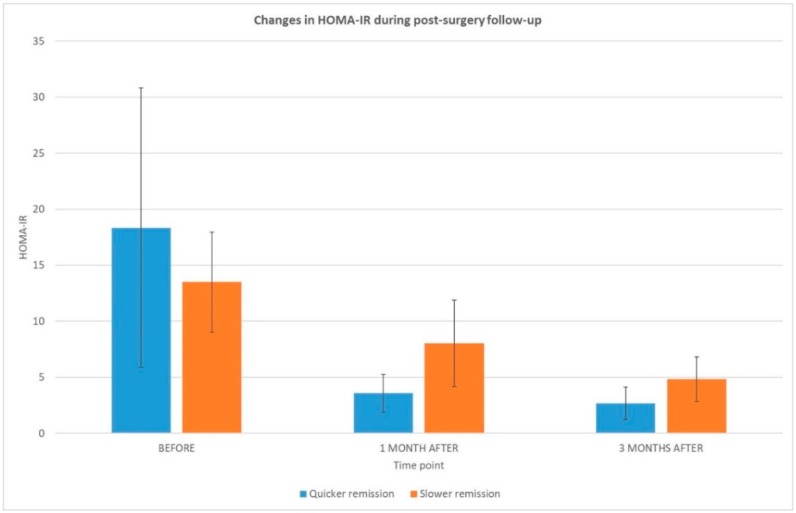
Changes in HOMA-IR (with its standard deviation) during follow-up after the bariatric surgical procedure in the groups with quicker (**blue**) and slower (**orange**) diabetes remission.

In the present study, accurate clinical, biochemical and metabolic data were analyzed to search for pre-operative and post-operative indicators of a higher remission tempo of T2DM after bariatric surgery. To recognize patients with quicker and slower rates of T2DM remission, a level of post-surgery HOMA-IR reduction was used. Three months HOMA-IR reduction has been previously proposed as the main predictor of T2DM complete remission [12]. Twenty obese patients with type 2 diabetes who underwent laparoscopic sleeve gastrectomy were included in the presented study. The selected study participants were divided into two groups (quicker (*n* = 11) and slower (*n* = 9) T2DM remission), depending on the rate of HOMA-IR reduction. The reduction of HOMA-IR during three months post-surgery in both groups of patients is depicted in Figure 1.

### 2.1. Bioclinical Outcome

At the beginning, the two established groups did not significantly differ in biochemical parameters. In both groups, laparoscopic sleeve gastrectomy evoked similar weight loss and similarly affected other parameters like glucose, cholesterol (including LDL and HDL) and TG concentrations. Due to the experiment’s design between studied groups, significant differences in HOMA-IR reduction one and three months post-surgery were observed. Moreover, significantly different values for insulin and HOMA-IR were observed one month post-surgery. Consequently, we have focused on early (one month post-surgery) changes in plasma metabolome after bariatric interventions. Moreover, at this period of time, all patients had similar post-operative lifestyles which allowed us to get a possibly “clear” effect of surgery excluding, or with minimal impact from, other factors. Detailed patients’ characteristics are presented in Table 1.

### 2.2. Metabolomics

Metabolomics analyses were performed with the previously described [7] LC-MS and GC-MS methods for serum fingerprinting. This resulted in the detection of over 50 statistically significant and identified metabolites. 

**Figure 2:**
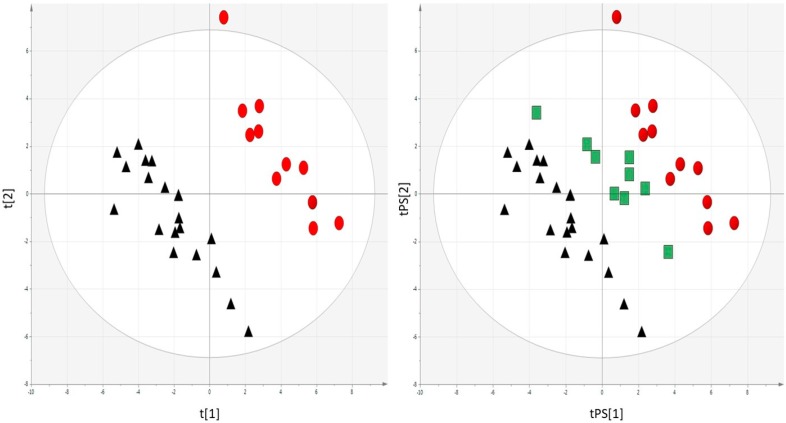
PLS-DA plots demonstrating the differences in the metabolome of the examined groups (▲—patients before the surgery, ●—one month after and quicker remission, ■—one month and slower remission). The left panel shows all patients clustered together before surgery and separated patients with quicker diabetes remission whose metabolic profiles differ after one-month post-surgery. A prediction of patients with slower remission by PLS-DA model built based on patients before and one-month post-surgery placed slower remission group exactly between the other groups (right panel). *R*^2^ = 0.947, *Q*^2^ = 0.434; Pareto Scaling, Log transformed data.

The multivariate statistical analysis confirmed differences in the metabolome of examined groups. Before the surgery, all patients clustered together; however, their metabolic profiles differed after one-month post-surgery. Interestingly, the prediction of patients with slower remission by PLS-DA model built based on patients before and one-month post-surgery (Figure 2, left panel) placed the slower remission group exactly between the other groups (Figure 2, right panel). 

In the present study, only several compounds (detected by LC-MS, Table 2) were changed in a similar way in both groups. These metabolites are 2-ketoisocaproic acid, glycerol and the following classes of lipids: phosphatidylcholines (PC), lysophosphatidylcholines (LPC), phosphatidylethanolamines (PE), lysophosphatidylethanolamines (LPE) and fatty acids (FA).

However, a much larger group of metabolites significantly changed one-month post-surgery in only one of the studied groups: quicker or slower T2DM remission (Table 3, 0 vs. 1 month columns). Again, in the case of LC-MS platform, significant metabolites can be classified mainly as PC, LPC, PE and LPE; additionally, changes in sphingomyelins (SM), fatty acids (FA) and fatty acid amides (FAAs) were observed. Some of them can be a component of biological membranes (PC, PE, SM); however, they can also be linked with type 2 diabetes (PC, PE, SM, FA), bacteria (PC, PE, SM) or oxidative stress (FAAs) [7,13]. The majority of them decreased post-surgery. This decrease was usually observed in the group with quicker diabetes remission. Additionally, some of them segregated in the studied groups before surgery or one-month after (Table 3, Quicker vs. Slower remission columns). It shows that a group with quicker remission already had lower levels of some lipids before the surgery or this level decreased quicker after the bariatric intervention. Only two metabolites increased after the surgery-PC 22:5/16:0 (FC = 1.27, *p* < 0.05) and SM 33:2 (FC = 1.32, *p* < 0.001) and this effect was observed only in the group with slower T2DM remission. Although the general tendency observed was a decrease of serum lipids after bariatric surgery, an increase of selected lipids has been already reported in other studies, including ours [7,8].

In the case of GC-MS results: amino acids, organic acids, sugars, metabolites related to gut microbiota metabolism and others (Table 4) were found significantly segregating patients with quicker and slower T2DM remission.

### 2.3. Correlation Analysis

Spearman’s rank correlation analysis of biochemical, clinical and metabolomics results was performed. Intensities of metabolites at 0 and one-month post-surgery were used for the correlation analysis. As a result, statistically significant correlations between carnitines, α-HB, β-HB, citric acid, fatty acids, glycerol, taurine, amino acids (e.g., l-valine, l-isoleucine, l-leucine, l-tyrosine, l-threonine, l-methionine) and others with parameters like weight, BMI, HOMA-IR and its decrease, glucose and insulin concentrations, cholesterol (including LDL and HDL fractions), glycated haemoglobin and the % excess weight loss were observed. Selected results are listed below and depicted on Figure 2.

Carnitines correlated mainly with HOMA-IR decreased (l-acetylcarnitine: r_S_ = 0.738, *p*-value = 0.046; l-stearoylcarnitine: r_S_ = 0.761, *p* = 0.037; linoleylcarnitine: r_S_ = −0.881, *p* = 0.007; elaidic carnitine: r_S_ = −0.833, *p* = 0.015) in the group with quicker remission and with HOMA-IR (linoleylcarnitine and HOMA-IR before: r_S_ = 0.857, *p* = 0.011; elaidic carnitine and HOMA-IR before: r_S_ = 0.786, *p* = 0.023; linoleylcarnitine and HOMA-IR 1 month after: r_S_ = −0.810, *p* = 0.022; elaidic carnitine and HOMA-IR 1 month after: r_S_ = 0.762, *p* = 0.037) and glucose level (linoleylcarnitine and glucose concentration one month after surgery: r_S_ = 0.826, *p* = 0.017; elaidic carnitine and glucose concentration after one month: r_S_ = 0.778, *p* = 0.030) in the group with slower T2DM remission. There was a correlation of α-HB with cholesterol (r_S_ = 0.762, *p* = 0.037), glucose (r_S_ = 0.838, *p* = 0.013) and insulin concentration (r_S_ = 0.786, *p* = 0.028), diabetes duration (r_S_ = 0.783, *p* = 0.026), taurine (r_S_ = 0.905, *p* = 0.005), citric acid (r_S_ = 0.738, *p* = 0.046), 2-ketoisocaproic acid (r_S_ = −0.738, *p* = 0.049) and l-valine (r_S_ = 0.738, *p* = 0.037) in the group with slow remission and with weight (r_S_ = 0.814, *p* = 0.018), oleamide level (r_S_ = −0.810, *p* = 0.022), 2-ketoisocaproic acid (r_S_ = 0.905, *p* = 0.005) and l-valine (r_S_ = −0.762, *p* = 0.037) in the group with quicker remission. On the other hand, β-HB level correlated with glycated haemoglobin (r_S_ = −0.778, *p* = 0.030), oleic acid (r_S_ = 0.762, *p* = 0.037) and uric acid (r_S_ = 0.786, *p* = 0.028) in the group with rapid remission and with oleic acid (r_S_ = 0.738, *p* = 0.046), l-stearoylcarnitine (r_S_ = −0.857, *p* = 0.011), LDL concentration (r_S_ = 0.881, *p* = 0.007; r_S_ = 0.810, *p* = 0.022), taurine (r_S_ = 0.833, *p* = 0.015), l-stearoylcarnitine (r_S_ = −0.857, *p* = 0.011), 3-indolelactic acid (r_S_ = 0.857, *p* = 0.011), pyruvic acid (r_S_ = −0.857, *p* = 0.011), α-HB (r_S_ = 0.952, *p* = 0.001), l-valine (r_S_ = 0.738, *p* = 0.046) and tagatose (r_S_ = −0.833, *p* = 0.015) in the slower remission group. In the slower remission group, taurine correlated with BMI (r_S_ = 0.738, *p* = 0.046), HDL (r_S_ = −0.881, *p* = 0.007), LDL (r_S_ = 0.857, *p* = 0.011), HOMA-IR decrease (r_S_ = 0.738, *p* = 0.046), weight (r_S_ = 0.929, *p* = 0.002), l-valine (r_S_ = 0.762, *p* = 0.037), l-isoleucine (r_S_ = 0.738, *p* = 0.046), l-threonine (r_S_ = 0.905, *p* = 0.005), phenylalanine (r_S_ = 0.833, *p* = 0.015), fumaric acid (r_S_ = 0.857, *p* = 0.011) and ribose (r_S_ = 0.762, *p* = 0.037). In the rapid remission group, taurine correlated with HDL (r_S_ = 0.802, *p* = 0.021), glucose (r_S_ = 0.833, *p* = 0.015) and insulin (r_S_ = 0.762, *p* = 0.037) concentration, HOMA-IR (r_S_ = 0.762, *p* = 0.037), HOMA-IR decrease (r_S_ = −0.762, *p* = 0.037), weight loss (r_S_ = −0.862, *p* = 0.008), arachidonic acid (r_S_ = 0.857, *p* = 0.011), stearic acid (r_S_ = 0.833, *p* = 0.015) and 3-indolelactic acid (r_S_ = 0.762, *p* = 0.037). 

A performed correlation analysis confirmed the importance of metabolites detected during metabolomics analysis, their connection with biochemical and clinical parameters, as well as observed differences between compared groups. 

### 2.4. Metabolic Pathways Analysis

In the present study, MetaboAnalyst (Available online: http://www.metaboanalyst.ca/), a general tool for metabolomics analysis [14], was used to link obtained metabolomics data with potential modulation of biochemical pathways. This platform is a comprehensive web-based tool suite designed to help users easily perform data analysis, visualization and functional interpretation [15].

**Figure 3:**
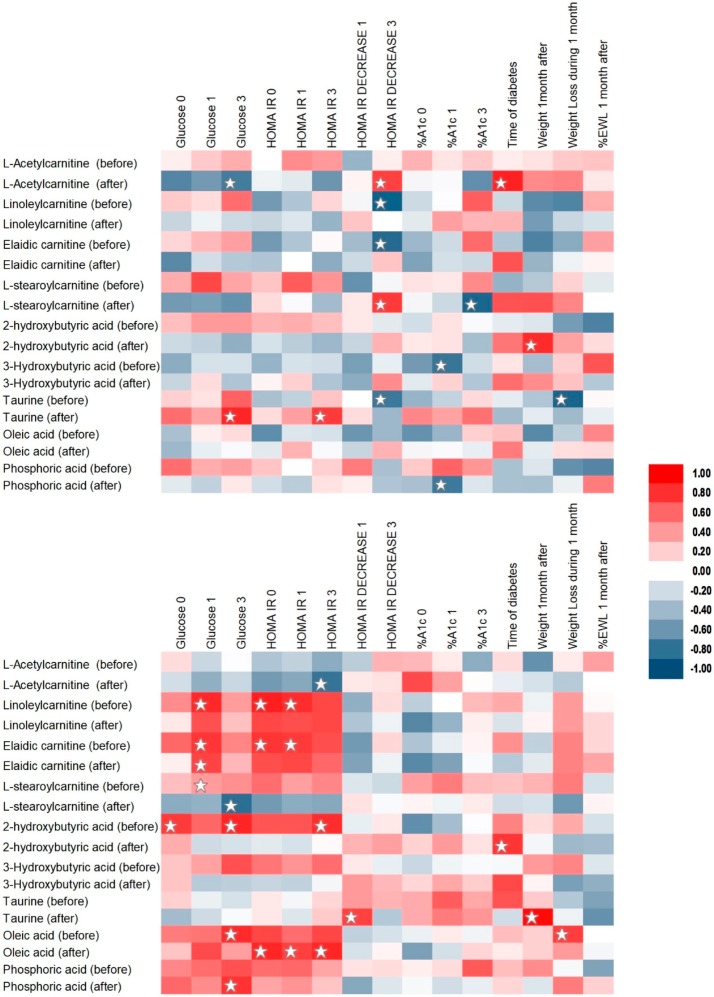
Selected Spearman correlation analysis results presented as heat maps for quicker (**top**) and slower (**bottom**) T2DM remission groups. Statistically significant correlations are marked with ☆. Time points: 0—before the surgery, 1—one month after, 3—three months after; Metabolites: “before”—before the surgery, “after”—one month post-surgery.

Results from MetaboAnalyst showed that bariatric surgery induced changes in numerous metabolites that are involved in many biochemical pathways; however, arranged pathways are different or have different impact in both compared groups (Figure 3 and Table 5). In the group with quicker remission, pathways linked to an energetic process, such the citrate cycle (TCA cycle) (*p*-value = 0.01, impact 0.08) or taurine metabolism (*p* = 0.01, impact 0.36), seems to be the most important. Synthesis and degradation of ketone bodies, which can be a link between energetic processes and diabetes, was also significantly altered (*p* = 0.047, impact 0.00). It is interesting to note the presence and importance of pathways related to microbiota-butanoate metabolism (*p* = 0.003, impact 0.02), propanoate metabolism (*p* = 0.03, impact 0.00), and alanine, aspartate and glutamate metabolism (*p* = 0.014, impact 0.06). It can suggest mitochondria and microbiota activity alterations [16,17,18]. On the other hand, molecular alterations in the group with slower diabetes remission seem to be focused around amino acids metabolism. Important alterations in such pathways as BCAA biosynthesis (*p* = 0.00001, impact 0.07) and degradation (*p* = 0.001, impact 0.06), phenylalanine, tyrosine and tryptophan biosynthesis (*p* = 0.00418, impact 0.01), and glycine, serine and threonine metabolism (*p* = 0.02, impact 0.10) were found. Additionally, other pathways with lower impact, aminoacyl-tRNA biosynthesis (*p* = 0.0000001, impact 0.00), propanoate metabolism (*p* = 0.0087, impact 0.00) and nitrogen metabolism (*p* = 0.001, impact 0.00) were listed. 

## 3. Discussion

In recent years, laparoscopic sleeve gastrectomy has become one of the most commonly used bariatric procedures, especially for morbid obesity. Although its role in diabetes treatment is still discussed; it is well documented that it leads to significant improvement in biochemical glucose homeostasis and can be a treatment method for patients with glucose metabolism abnormalities [10]. There are numerous hypotheses concerning potential causes of those effects, however, and many aspects of the metabolic effects of bariatric surgery remain unanswered [2,11,19]. In the meantime, an understanding of the metabolic changes induced by bariatric surgery may lead to new treatment strategies for obesity and related co-morbidities. 

In our previous study, we presented the hypothesis that bariatric procedures in a semi-annual perspective lead to similar clinical effects, like weight loss or diabetes remission; however, molecular mechanisms involved in those outcomes varied dependently on bariatric procedure [7]. Instead, the study presented here shows that molecular mechanisms can also differ inside one surgery group. Here, these changes were demonstrated in the various pace of type 2 diabetes recovery. The performed metabolomics analyses suggest that mitochondria can play a crucial role in the process of bariatric surgery-induced T2DM recovery. However, the presence of microbe’s related metabolites suggests significant alteration in gut microbiota activity and/or compositions. Additionally, an interesting finding was the detection of a group of metabolites which can be matched with changing mitochondria-microbes relation. This kind of connection could be, according to our knowledge, the first report on mitochondria influence and microbiota relationship to type 2 diabetes remission in the aspect of bariatric surgery. However, this effect should be confirmed by additional analyses. Performed correlation and pathways analyses have confirmed the crucial role of mitochondria as well as gut microbiota influence on T2DM recovery. 

Mitochondrial metabolism is essential in maintaining physiological function in human cells, e.g., performing fatty acid oxidation and providing adenosine triphosphate (ATP) [19]. They are also a major producer of reactive oxygen species and reactive nitrogen species. Mitochondria are involved in numerous essential cell functions, e.g., energy metabolism, apoptotic pathways and steroid hormone synthesis. However, their function is reduced in insulin-responsive tissues in obesity and T2DM. Notably, insulin signaling was shown to impact mitochondrial DNA, protein synthesis and affect mitochondrial respiration and ATP production. No consensus has been reached on whether insulin resistance is a result of reduced mitochondrial density and whether it is the cause or consequence of mitochondrial dysfunction [19]. It was reported that destabilized in T2DM and obesity mitochondrial metabolism can be influenced by bariatric surgery. For example, lipid oxidation was higher in the diabetic group after bariatric surgery, which can suggest a different regulation of mitochondrial function in response to bariatric intervention in comparison to patients with normal glucose tolerance. Animal studies have shown a relation between Roux-en-Y gastric bypass and the mitochondrial fusion protein, Mfn1 levels, PGC1α and NRF1 expression, citrate synthase activity and mitochondrial respiration, which has shown an association between RYGB and improved mitochondrial dynamics [19,20]. Detected significant alterations in lipids, carnitines, amino acids, α-HB and β-HB and citric acid levels suggest meaningful modulation of mitochondria activity. Elevated levels of L-acetylcarnitine (FC = 2.24, *p* < 0.05) and linoleylcarnitine (FC = 1.45, *p* < 0.01) were observed in the group with quicker T2DM remission, increased level of elaidic carnitine was observed in both groups (FC = 1.55, *p*-value < 0.01 in quicker and FC = 1.46, *p* < 0.05 in slower remission group), while the level of L-stearoylcarnitine decreased in the slower remission group (FC = 0.6, *p* < 0.05). Carnitines are involved in the mitochondrial transport of fatty acid and are of critical importance for maintaining normal mitochondrial function. L-carnitine and its esters help to reduce oxidative stress, whereas acetyl-L-carnitine increases insulin sensitivity and glucose tolerance [21,22,23]. Fatty acid metabolism is dependent on mitochondria function; however, they may also be affected by impaired muscle BCAA catabolism. Plasma BCAAs are elevated in obese insulin resistant humans and their reduction was observed after bariatric surgery or weight loss [24]. Interestingly, during the first month of post-surgery, we observed a significant decline of amino acids only in the slower responders group. Nevertheless, there is a complex interplay between BCAA and lipids metabolism as well as the tricarboxylic acid (TCA) cycle, which leads to insulin resistance and T2DM development [25]. Considering the differences in the change of citric acid and fumaric acid, as well as α-HB and β-HB, the TCA cycle is also differentially affected in quicker and slower responders. Citric acid was found dramatically increased in the group with quick diabetes improvement (FC = 2.75, *p* < 0.05) and it was noticeably higher after the surgery in this group (FC = 2.48, *p* < 0.01). Citrate is an important substrate in cellular energy metabolism—it inhibits and induces important strategic enzymes located at the entrance and/or at the exit of glycolysis, TCA cycle, gluconeogenesis and fatty acids synthesis. Citric acid is synthesized in mitochondria from acetyl-CoA and oxaloacetate (OAA) and it becomes a substrate in the TCA cycle. Its subsequent complete oxidation provides the major source of cellular ATP production. Beyond the classical role as a metabolic regulator, its role in inflammation, cancer, insulin secretion, histone acetylation, disorders and diseases has been highlighted [26]. Fumaric acid is likewise a metabolite connected with the Krebs cycle. This dicarboxylic acid is a precursor to L-malate in the TCA cycle. In our study, its elevated level was observed in the group with rapid diabetes remission (FC = 1.91, *p* < 0.05). Uniquely, in the group with quick diabetes improvement, a noticeable increase in the level of α-hydroxybutyric acid (FC = 1.69, *p* < 0.05) and β-hydroxybutyric acid (FC = 7.32, *p* < 0.01) was observed. α-HB is an organic acid derived from α-ketobutyrate and is metabolized to propionyl-CoA and carbon dioxide. It was reported as an early marker for insulin resistance and impaired glucose regulation. The 2-hydroxybutyric acid level can elevate at least in two conditions—elevation of hepatic glutathione stress and elevation of the nicotinamide adenine dinucleotide (NADH)/nicotinamide adenine dinucleotide (NAD+) ratio due to increased lipid oxidation [13]. β-hydroxybutyric acid is the ketone body and energy carrier from adipocytes during fasting or exercise. Additionally, its downstream metabolism products have signaling activities. Metabolism of β-HB increases cellular levels of acetyl-CoA and reduces levels of succinyl-CoA and NAD+. These can further increase mitochondrial protein acetylation and reduce mitochondrial protein succinylation, potentially regulating the function of many metabolic enzymes. The relative sparing of cytoplasmic NAD levels with a utilization of 3-hydroxybutyric acid rather than glucose can alter the activity of NAD-dependent enzymes such as sirtuins. Finally, β-HB might increase histone acetylation and alter gene expression by generativity acetyl-CoA in mitochondria, which can be transported into the nucleus via the citrate shuttle to serve as a substrate for histone acetyltransferases. These regulatory functions have important implications for the pathogenesis and treatment of metabolic diseases including type 2 diabetes [27]. Therefore, significant alterations of above mentioned metabolites suggest relation between the rate of T2DM recovery after bariatric surgery and energy metabolism.

**Figure 4:**
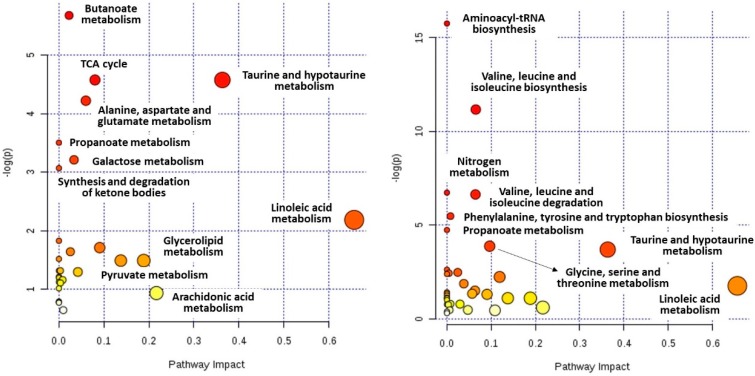
Summary of Pathway Analysis for group with quicker (**left**) and slower (**right**) diabetes remission.

Recent advances have also highlighted the impact of the gut microbiota on human health [18]. The microbiota offer many benefits to the host; however, gut dysbiosis (altered gut bacterial composition) was identified in overweight and obesity and marked as being associated with metabolic alterations such as insulin resistance, low-grade inflammation or adipocyte hypertrophy [28]. Data from human and animal studies have demonstrated that microbiota composition is modified after bariatric interventions. Bariatric surgery induces changes in the digestive tract anatomy and hormonal status; ingested nutrients and this modulation may impact the microbiota composition [29]. Detected alterations in the level of amino acids (especially BCAA), taurine, lactic acid, glycerol, α-HB and β-HB and the contribution of butanoate metabolism, propanoate metabolism, alanine, aspartate and glutamate metabolism pathways (Figure 4, Table 5) suggest microbe’s contribution in observed post-surgical molecular changes, including modulations related to T2DM remission.

In our study, the rapid remission group presented a base reduced taurine level in comparison to the slow remission group (FC = 0.43, *p* < 0.05). Taurine has a wide range of cytoprotective actions and inhibits the development of diet-related diseases such as diabetes and non-alcoholic fatty liver disease. The principal mechanism for its appearance in the intestine is secretion of taurine-conjugated bile acids followed by microbial deconjugation and the release of taurine. Its re-absorption is low, since taurine transporters are mostly located upstream in the small intestine; however, the *Lactobacillus* species are effective at deconjugating bile acids, specifically those conjugated to taurine. Additionally, bile acid deconjugation by *Lactobacillus* can be further stimulated by high glycolytic activity. Increased deconjugation of bile acids promotes the elimination of taurine by depleting endogenous taurine pools, which may reduce protection against complications of diabetes [30]. Among other microbe related metabolites, lactic acid can be mentioned. It decreased after one month-post surgery in the group with quicker remission (FC = 0.58, *p* < 0.05) and was lower in this group compared to before surgery (FC = 0.47, *p* < 0.05). Studies on animals have shown that specific strains of a lactic acid bacterium can be expected to be beneficial for the management of type 2 diabetes [31]. Lactate level reflects anaerobic metabolism and is a measurement of mitochondrial dysfunction from low mitochondrial oxidative capacity. Elevated lactate levels were observed in obese individuals and correlated positively with blood pressure and insulin resistance. Moreover, it was reported that lactate levels correlated inversely with the mitochondrial DNA copy number in PBMCs, suggesting the association between mitochondrial dysfunction and lactate levels [32]. The fermentation product 2,3-butanediol, which can be produced by fermenting bacteria, was reported as a factor which has a role in bacterial virulence, abundance and biofilm formation. It can also result in dynamic shifts in microbiota diversity [33]. In the presented study, a lower base level of the 2,3-butanediol derivative was observed in the group with rapid remission (FC = 0.41, *p* < 0.01). An interesting finding was an elevated level of phosphoric acid in the group with quicker diabetes remission (FC = 4.55, *p* < 0.05). It was reported that it showed antimicrobial activity and this action can be connected with increasing the hydrogen-ion concentration in the microorganism which cannot survive these conditions [34]. Microbe related metabolite is also an oxotetradecenoic acid, which was described as a component of bacterial lipopolysaccharide. Elevated levels (FC = 2.06, *p* < 0.05) were observed in the quicker remission group. Additionally, alterations in sulfate-containing metabolites, carnitines, amino acids (tryptophan, phenylalanine, tyrosine) or β-hydroxybutyric acid can be linked with gut microbes and their alterations [8].

Several studies have previously reported a bidirectional interaction between microbiota quality and mitochondrial function [16,17,18] and selected metabolites or pathways taking part in those interactions were observed in the described analyses. It is intriguing to note the presence of nitrogen related metabolites and the influence of nitrogen metabolism in the pathways analysis; however, its role can also be explained. The mitochondria-microbiota cross-talk is intriguing because mitochondria share many structural and functional features with the prokaryotic world. Moreover, this inter-talk may be crucial for human health—obesity, diabetes mellitus and Crohn’s disease are associated with microbiota composition and different levels of *Bacteroides* and Firmicutes phyla [16,18]. Recent studies have highlighted the importance of a few key microbiome metabolites. The short-chain fatty acids (SCFA), especially butyrate and propionate, urolithins, lactic acid and lactate, selected carnitines, TCA related metabolites, lipopolysaccharides, flagelin, lipoteichoic acid, amino acids, fatty acids, dinitrophenol, hydrogen sulfide, nitric oxide, l-arginine and thiol can be mentioned here. [17,18] Few reports have described how commensal or pathogenic microbiota can modulate mitochondrial function. In response to the microbe’s activity, mitochondria can react in a few ways. Its main mechanisms are the regulation of energy production, alteration of redox balance, regulation of immune reactions by attenuating TNFα-induced and inflammation-induced oxidation, which leads to mitochondrial dysfunction. On the other hand, mitochondria can modify microbiota composition through a production of reactive oxygen species and reactive oxygen nitrogen species, induction of the secretion of immune cells and enterochromaffin cells, modulation of gut functions (intestinal barrier function, mucosal immune response) mitochondrial genetic variants and heteroplasmy. An association between polymorphism of mitochondrial genes or D-Loop region in the mitochondrial genome and specific gut microbiota compositions was previously reported [16]. This can suggest that patients differ in gut microbiota compositions even before the surgery and that composition together with modulated mitochondrial activity can be an important factor in different T2DM remission tempos. The concentration of ROS directly correlated with the activity of the electron transfer chain, depending on its level, can induce differentiation or proliferation of a cell, cytokine release or apoptosis [18]. Additionally, increased ROS production may lead to lipid peroxidation. Interestingly, during the first month of post-surgery, we observed significant changes in oxidized fatty acids (Table 3), though only in the quicker remission group. The release of pathogenic lipopolysaccharydes, flagelin, lipoteichoic acid, lipoprotein or other toxins can generate signals which influenced specific receptors leading to an inflammatory response [18]. Some tend to increase ROS production by the mitochondrial respiratory chain. Activation of the immune system generally increases ATP production in lymphocytes, usually due to high glycolytic activity and mitochondrial fatty acid oxidation related with a reduction of electron transfer chain gene expression. Related to the Warburg effect, high glycolytic activity leads to a high production of different precursors involved in the biosynthesis of nicotinamide adenine dinucleotide phosphate (NADPH), amino acids, nucleotides and fatty acids. Additionally, stimulation of lymphocyte B causes upregulation of glycolysis and oxidative phosphorylation [18]. Dinitrophenol increases ROS production and affects epithelial barrier function leading to structurally abnormal mitochondria [17,18]. Due to the degradation of sulfur amino acids in the gut, some bacteria species can produce a large amount of hydrogen sulfide. Its elevated concentration inhibits cytochrome oxidase—one of the major complexes of the mitochondrial respiratory chain. Nitric oxide is produced by the host during inflammation due to L-arginine conversion or nitrate reduction, which is suppressed energy metabolism by reducing acetyl-CoA production. Thus, the presence of microbiota-related factors may affect mitochondria activity and ROS production [18]. The aforementioned butyrate is known to be produced by bacteria. [8,17,35] It can enter the citric acid cycle to reduce NAD^+^ to NADH and can be used as a carbon source even in the presence of glucose. Thus, butyrate regulates mitochondrial activity and promotes the release of signaling hormones like GLP-1 related to lower food intake [18].

The obtained results indicate a relationship between gut microbiota and mitochondria modulation by bariatric surgery and consequent type 2 diabetes remission. All data and relationships presented above allowed us to conclude that a key player in diabetes remission can be mitochondria and their capability. However, gut microbiota can also be a determinative factor that can regulate their function. Observed findings are novel, and further research using specific microbiology and mitochondria-related measurements are needed for their confirmation and to give a full explanation of the exact molecular mechanisms.

## 4. Materials and Methods

### 4.1. Materials

#### 4.1.1. Study Participants

The study participants were selected from a cohort of 372 obese individuals who underwent laparoscopic sleeve gastrectomy in the 1st Clinical Department of General and Endocrine Surgery (Medical University of Bialystok, Bialystok, Poland). From this cohort, 20 patients with T2DM, but without post-surgery pharmacological anti-diabetes treatment, were selected. A majority of the experimental subjects had newly diagnosed T2DM and they did not receive anti-diabetic treatment before and after the surgery. Only a few patients from both groups were taking metformin before the surgery; however, the treatment was discontinued immediately after the performed surgery. Type 2 diabetes was recognized according to the American Diabetes Association’s criteria when fasting plasma glucose was ≥126 mg/dL, or 2 h plasma glucose in the 2 h-75 g OGTT ≥ 200 mg/dL, or HbA1c ≥ 6.5%. Based on biochemical and clinical parameters, with special impact to HOMA-IR reduction, patients were divided into two groups with a quicker or slower rate of type 2 diabetes remission. Both groups were matched in age, sex, BMI, diet and other basic parameters. Detailed characteristics of the study groups are presented in Table 1. Metabolomics analyses were performed on fasting serum samples obtained before and one month after surgery. The study was performed with the approval of the Ethics Committee at the Medical University of Bialystok (R-I-002/531/2013, 28 November 2013), upon written consent from all participants.

#### 4.1.2. Samples Collection

Venous blood was drawn from participants in the fasting state into syringes containing a clotting activator (serum) or NaF/Na_2_EDTA (plasma). To obtain the serum, the whole blood was allowed to clot by leaving it undisturbed at room temperature for 30 min. Serum and plasma were obtained by centrifugation at 1300× *g* for 30 min in 4 °C. Aliquots of the samples were stored at −80 °C until analysis.

#### 4.1.3. Chemicals

Purified water was obtained using the Milli-Qplus185 system (Millipore, Billerica, MA, USA). LC–MS grade methanol (MeOH), acetonitrile (ACN), formic acid and LC grade ethanol (EtOH), standard mix for GC–MS, containing grain fatty acid methyl ester mixture (C8-C28), 2-propanol and analytical grade heptane were purchased from Sigma-Aldrich ChemieGmbH (Steinheim, Germany). C18:0methyl ester and *N*,*O*-bis(trimethylsilyl) trifluoroacetamide with 1% trimethylchlorosilane were obtained from Pierce Chemical Co (Rockford, IL, USA). Silylation grade pyridine was purchased from VWR International BHDProlabo (Madrid, Spain). The API-TOF reference mass solution kit (G1969-850001) and tuning solutions, ESI-L low concentration tuning mix (G1969-85000) and ESI-TOF Biopolymer Analysis referencemasses (G1969-850003) were purchased from Agilent Technologies (Santa Clara, CA, USA).

### 4.2. Methods

#### 4.2.1. Biochemical Measurements

Plasma glucose concentrations were analyzed by a hexokinase method (Roche Diagnostics International Ltd., Rotkreuz, Switzerland) and plasma level of HbA1c was measured by the HPLC method (Bio-Rad VARIANT). During these measurements, external and internal quality controls were positioned within the allowed ranges.

The HOMA-IR index was estimated using the following formula:

HOMA-IR = fasting insulin (mIU/L) × fasting glucose (mmol/L)/22.5

#### 4.2.2. Metabolic fingerprinting by LC-MS and GC-MS

Metabolic fingerprinting was accomplished on LC−MS (6550, Agilent Technologies, Santa Clara, CA, USA) and GC−MS (5975C, Agilent Technologies, Santa Clara, CA, USA) platforms. Serum samples preparation, analysis and data treatment were performed according to the previously described protocols [7].

The Identification of metabolites detected by LC-MS was performed only for statistically significant metabolic features. The identity of compounds was confirmed by LC-MS/MS using a QTOF (model 6550, Agilent). Obtained MS/MS spectra were compared with the spectral data of reference compounds (HMDB, METLIN, LIPIDMAPS through CEU mass mediator), and product ions of commercially (Sigma) available reagents with those obtained in real samples. Phospholipids, lysophospholipids and sphingomyelins were confirmed with recently described characteristic fragments [36].

#### 4.2.3. Statistical Analysis

Statistical analysis was performed to identify the differences in serum metabolites between two groups of patients established based on the information about T2DM remission rate. Primarily, serum samples obtained before surgery were compared in order to determine whether prior surgery metabolic profiles between patients with quicker and slower T2DM remissions were different. The other comparison was performed to find exact metabolic changes during the month after the surgery in patients with faster and slower remissions of T2DM. For the comparison of patients before surgery, a selection of statistically significant metabolites was performed using a *t*-test or Mann-Whitney U-test (depending on the normality of data distribution). To compare patients in time (before and after the surgery), depending on the data distribution, a paired *t*-test or Wilcoxon signed rank test was used. The normality of data distribution was assessed using the Shapiro–Wilk test. Equality of variance for compared groups was tested by an F-test. The *t*-test, paired *t*-test and F-test were calculated with Excel (Microsoft Corporation, Redmond, WA, USA) while the U-test, Wilcoxon signed rank test, Shapiro-Wilk tests and Spearman’s rank correlation coefficient were performed with MATLAB 7.10 R2010a (MathWorks Inc., Natick, MA, USA).

## 5. Conclusions

In this study, we presented that a modified level of carnitines, lipids, amino acids and α- and β-hydroxybutyric acids suggest a major role of mitochondria activity in the T2DM remission process. Additionally, some of the observed metabolites suggested that post-surgery modulation of gut microbiota composition may correlate with diabetes’ recovery tempo. Additional analyses confirmed a relationship between biochemical and clinical parameters and aforementioned metabolites, thereby highlighting the role of mitochondria and microbes in the rate of T2DM remission.

According to our knowledge, the correlation between bariatric surgery evoked mitochondria and gut microbiota modulation and type 2 diabetes remission has been presented here for the first time. More investigation is needed to confirm and explore these findings. Therefore, we believe that the presented results indicate novel directions of research. Examination of mitochondria modulation and gut microbiota activity after bariatric surgery may help to reveal the mechanism by which this type of surgery leads to T2DM remission.

## Figures and Tables

**Table 1:** Characteristics of study participants.

Time Frame	Biochemical and Clinical Parameters	Quicker Remission	Slower Remission	*p*-Value
	Age (years)	47 ± 10.3	51 ± 11.4	ns
	Sex–F/M	4/7	4/5	-
	Body Mass Index (BMI) before	49 ± 4.5	51 ± 8.3	ns
	BMI 1 month after	42 ± 3.5	46 ± 8.1	ns
	BMI 3 months after	37 ± 3.6	43 ± 7.6	ns
	Excess weight loss (EWL, %) 1 month after	21 ± 5.9	18 ± 4.8	ns
	Excess weight loss (EWL, %) 3 months after	29 ± 9.8	29 ± 7.4	ns
	Weight loss after 1 month (kg)	16 ± 4.7	15 ± 2.8	ns
	Time of type 2 diabetes (T2DM) lasting (months)	30 ± 59.3	24 ± 16.5	ns
**Before surgery**	Glucose (mg/dL)	158 ± 62.6	153 ± 36.1	ns
	Insulin (pmol/L)	46 ± 39.4	36 ± 13.8	ns
	HOMA-IR	18.3 ± 12.5	13.5 ± 4.5	ns
	Cholesterol (mg/dL)	198 ± 43.4	186 ± 27.7	ns
	LDL (mg/dL)	147 ± 43.4	114 ± 29.2	ns
	HDL (mg/dL)	50 ± 22.8	42 ± 6.6	ns
	TG (mg/dL)	150 ± 55.2	154 ± 51.5	ns
	A1c (%)	6.88 ± 1.3	6.26 ± 0.7	ns
**1 month post-surgery**	Glucose (mg/dL)	102 ± 20.3	118 ± 22.0	ns
	Insulin (pmol/L)	13 ± 7.7	26 ± 11.1	0.011
	HOMA-IR	3.6 ± 1.7	8.0 ± 3.9	0.029
	HOMA-IR decrease (%)	76 ± 8.9	42 ± 16.2	0.000
	Cholesterol (mg/dL)	190 ± 43.5	197 ± 28.7	ns
	LDL (mg/dL)	126 ± 38.0	135 ± 30.0	ns
	HDL (mg/dL)	48 ± 33.5	59 ± 43.9	ns
	TG (mg/dL)	130 ± 45.4	149 ± 75.5	ns
	A1c (%)	6.3 ± 1.0	5.9 ± 0.4	ns
**3 months post-surgery**	Glucose (mg/dL)	99 ± 17.7	108 ± 11.3	ns
	Insulin (pmol/L)	10 ± 6.1	18 ± 8.5	ns
	HOMA-IR	2.67 ± 1.5	4.82 ± 2.0	ns
	HOMA-IR decrease (%)	80 ± 12.3	63 ± 13.9	0.011
	Cholesterol (mg/dL)	184 ± 46.8	188 ± 25.3	ns
	LDL (mg/dL)	128 ± 37.8	132 ± 35.4	ns
	HDL (mg/dL)	51 ± 19.5	47 ± 11.8	ns
	TG (mg/dL)	125 ± 37.8	139 ± 28.7	ns
	A1c (%)	5.8 ± 0.6	6.5 ±1.3	ns

**Table 2:** Metabolites altered similarly before and one month after the surgery in patients with quicker and slower type 2 diabetes (T2DM) improvement detected with liquid chromatography coupled with mass spectrometry (LC-MS).

Metabolite	Quicker Remission	Slower Remission
Lyso PC 14:0 (+)	0.54 **	0.56 *
Lyso PC 20:5*sn*-2 (+)	0.50 ****	0.61 *
Lyso PC 20:3 (+)	0.41 ****	0.61 *
Lyso PC 20:5*sn*-1 (+)	0.50 ****	0.61 *
PC 30:0 (+)	0.49 **	0.60 *
PC 32:1 (+)	0.56 **	0.58 **
PC 34:4 (+)	0.48 **	0.54 *
PC 40:5 (+)	0.42 **	0.51 *
Lyso PE 18:1 (−)	0.72 *	0.72 *
Lyso PE 20:5 (+/−)	0.54 **	0.55 *
PE 16:0/20:5 (−)	0.52 **	0.56 *
arachidonic acid (−)	1.34 **	1.31 **

Alterations are presented as fold change (FC). PC: phosphatidylcholines; PE: phosphatidylethanolamines. (+)/(−)—metabolite identified in positive or negative ESI mode, respectively; ns—respectively; ns—non-significant or significant but not identified; * *p* ≤ 0.05; ** *p* ≤ 0.01; *** *p* ≤ 0.001; **** *p* ≤ 0.0001.

**Table 3:** Metabolites discriminating quicker and slower T2DM remission groups detected with LC-MS.

Metabolite	0 vs. 1 Month		Quicker vs. Slower Remission	
	Quicker Remission	Slower Remission	Before Surgery	1 Month Post-Surgery
l-Acetylcarnitine(+)	2.24 *	ns	ns	2.32 *
Linoleylcarnitine (+)	1.45 **	ns	ns	ns
Elaidic carnitine (+)	1.55 **	1.46 *	ns	ns
l-stearoylcarnitine (+)	ns	0.60 *	ns	ns
Lactic acid (−)	0.58 *	ns	ns	ns
Uric acid (−)	1.26 *	ns	ns	ns
Oleic acid (−)	1.40 **	1.39 *	ns	0.67 *
Oleamide (+)	ns	0.62 ****	ns	ns
Stearamide (+)	ns	0.75 *	ns	ns
Lyso PE 16:0 (−)	ns	ns	0.56 *	ns
Lyso PE P-16:0 (+)	ns	ns	0.75 *	ns
Lyso PE 18:0 (+/−)	0.72 *	ns	066 *	0.65 *
Lyso PE 18:1 (+/−)	ns	ns	0.66 *	0.63 *
Lyso PE 18:2 (+/−)	0.61 **	ns	ns	0.63 *
Lyso PE 20:0 (−)	ns	ns	ns	0.61 *
Lyso PE 20:3 (+/−)	0.51 *	ns	0.63 *	0.63 *
Lyso PE 20:4 (−)	ns	ns	ns	0.71 *
Lyso PE 22:5 (−)	ns	ns	0.55 *	0.56 *
PE 18:1/16:0 (−)	ns	0.74 *	ns	ns
PE 18:2/16:0 (−)	0.61 *	ns	ns	ns
PE 16:0/20:5 (−)	ns	ns	0.70 *	ns
PE 18:0/18:2 (−)	0.50 *	ns	ns	ns
PE 18:0/20:3 (−)	0.54 ****	ns	ns	ns
Lyso PC 14:0 (−)	0.65 *	ns	ns	0.57 *
Lyso PC 15:0 (−)	ns	ns	0.75 *	0.69 *
Lyso PC 16:0 (−)	ns	ns	0.64 *	ns
Lyso PC 16:1 (+)	0.66 *	ns	ns	ns
Lyso PC 17:1 (+)	ns	ns	0.71 *	ns
Lyso PC 18:0 (+/−)	0.71 **	ns	0.72 *	0.59 *
Lyso PC 18:2 (+/−)	0.73 *	ns	ns	0.69 *
Lyso PC 20:1 (−)	ns	ns	0.76 *	ns
Lyso PC 20:2 (+/−)	0.70 **	ns	0.70 *	ns
Lyso PC 20:3 (+/−)	0.60 *	ns	ns	ns
Lyso PC 22:4 (+)	0.70 *	ns	ns	ns
Lyso PC 22:5 (+)	0.61 **	ns	1.50 *	ns
PC 30:0 (+)	ns	ns	ns	0.64 *
PC 32:2 (+)	0.49 **	ns	ns	ns
PC 34:3 (+)	0.56 **	ns	ns	ns
PC 18:2/17:0 (−)	0.68 **	ns	ns	ns
PC 38:5 (+)	0.27 *	ns	ns	ns
PC 22:5/16:0 (−)	ns	1.27 *	ns	ns
SM 33:2(−)	ns	1.32 ***	ns	ns
C14:1 sphingolipid (+)	0.68 ****	ns	0.49 *	ns
Sphingosine (+)	ns	ns	ns	1.40 *
Choline (+)	ns	0.70 *	0.49 *	ns
Lueucine/isoleucine (+)	ns	0.62 ****	ns	ns
Piperine (+)	ns	0.58 *	0.48 *	ns
Oxotetradecenoic acid or hydroxytetradecenoic acid (−)	2.06 *	ns	ns	ns
Hydroperoxyoctadecadienoic acid (−)	2.06 *	ns	ns	ns
Succinyldiaminopimelic acid (−)	ns	0.75 ****	ns	ns
Hydroxyandrostanone sulfate (−)	1.28 ****	1.35 *	ns	ns
Androsterone sulfate (−)	1.31 ****	ns	ns	ns
Hydroperoxylinoleic acid (−)	0.55 *	ns	ns	ns
Taurine (−)	ns	ns	0.43 *	ns

Alterations are presented as fold change (FC). (+)/(−)—metabolite identified in positive or negative ESI mode, respectively; ns—non-significant or significant but not identified; * *p* ≤ 0.05; ** *p* ≤ 0.01; *** *p* ≤ 0.001; **** *p* ≤ 0.0001. For lysophosphatidylcholines (LPCs) or lysophosphatidylethanolamines (LPEs), detected in both ion modes as well as for *sn*-1 and *sn*-2 isomers, an average FC is presented.

**Table 4:** Metabolites segregating quicker and slower T2DM remission groups detected with a gas chromatography mass spectrometry (GC-MS).

Metabolite	0 vs. 1 Month		Quicker vs. Slower Remission	
	Quicker Remission	Slower Remission	Before Surgery	1 Month Post-Surgery
2-hydroxybutyric acid	1.69 *	ns	ns	1.70 *
3-hydroxybutyric acid	7.32 **	ns	ns	4.76 **
2-ketoisocaproic acid	1.97 **	1.68 *	ns	ns
phosphoric acid	4.55 *	ns	ns	ns
fumaric acid	1.91 *	ns	ns	ns
l-(+) lactic acid	ns	ns	0.47 *	ns
citric acid	2.75 *	ns	ns	2.48 **
linoleic acid	ns	ns	ns	1.61 *
l-valine/norvaline	ns	0.76 **	ns	ns
l-leucine	ns	0.70 **	ns	ns
Isoleucine/norleucine	ns	0.76 *	ns	ns
l-threonine	ns	0.73 *	ns	ns
l-methionine	ns	0.68 **	ns	ns
Phenylalanine	ns	0.60 *	ns	ns
l-tyrosine	ns	0.62 **	ns	ns
l-tryptophan	ns	0.63 **	ns	ns
l-alanine	ns	ns	0.63 *	ns
*N*-methylalanine	1.81 *	ns	ns	ns
Glycerol	2.11 **	1.73 *	ns	ns
ribose	ns	0.44 *	ns	ns
d-mannose/d-allose	ns	0.69 *	ns	ns
Furanose (tagatose)	0.47 **	ns	ns	ns
2,3-Butanediol, 2TMS derivative	ns	ns	0.41 **	ns

Alterations are presented as fold change (FC); ns—non-significant, * *p* < 0.05, ** *p* < 0.01.

**Table 5:** Results (selected) from the Pathway Analysis.

Group	Pathway	*p*-Value	FDR	Impact
**Quicker remission**	Butanoate metabolism	3.43 × 10^−3^	2.74 × 10^−1^	0.02
	Citrate cycle	1.03 × 10^−2^	2.75 × 10^−1^	0.08
	Taurine and hypotaurine metabolism	1.03 × 10^−2^	2.75 × 10^−1^	0.36
	Alanine, aspartate and glutamate metabolism	1.47 × 10^−2^	2.94 × 10^−1^	0.06
	Propanoate metabolism	3.01 × 10^−2^	4.81 × 10^−1^	0.00
	Galactose metabolism	4.03 × 10^−2^	5.31 × 10^−1^	0.03
	Synthesis and degradation of ketone bodies	4.65 × 10^−2^	5.31 × 10^−1^	0.00
**Slower remission**	Aminoacyl-tRNA biosynthesis	1.44 × 10^−7^	1.15 × 10^−5^	0.00
	Valine, leucine and isoleucine biosynthesis	1.42 × 10^−5^	5.67 × 10^−4^	0.07
	Nitrogen metabolism	1.19 × 10^−3^	2.63 × 10^−2^	0.00
	Valine, leucine and isoleucine degradation	1.31 × 10^−3^	2.63 × 10^−2^	0.06
	Phenylalanine, tyrosine and tryptophan biosynthesis	4.18 × 10^−3^	6.69 × 10^−2^	0.01
	Propanoate metabolism	8.74 × 10^−3^	1.17 × 10^−1^	0.00
	Glycine, serine and threonine metabolism	2.07 × 10^−2^	2.37 × 10^−1^	0.10
	Taurine and hypotaurine metabolism	2.48 × 10^−2^	2.48 × 10^−1^	0.36

## Abbreviations

α-HB	α-hydroxybutyric acid/2-hydroxybutyric acid
β-HB	β-hydroxybutyric acid/3-hydroxybutyric acid
acetyl-CoA	acetyl coenzyme A
ATP	Adenosine triphosphate
BCAAs	branched-chain amino acids
BMI	body mass index
CLR	C-type lectin receptor
(%)EWL	(%) excess weight loss
FA	fatty acid
FAA	fatty acid amides
FC	fold change
FDR	false discovery rate procedure
GC	gas chromatography
HbA1c	hemoglobin A1c (glycated hemoglobin)
HDL	high-density lipoprotein
HOMA	homeostatic model assessment
IR	insulin resistance
LC	liquid chromatography
LDL	low-density lipoprotein
(L)PE	(lyso)phosphatidylethanolamine
(L)PC	(lyso)phosphatidylcholine
LPS	lipopolysaccharide
LSG	laparoscopic sleeve gastrectomy
*Mfn1*	Mitofusin 1
MS	mass spectrometry
NAD	nicotinamide adenine dinucleotide
NADH	nicotinamide adenine dinucleotide
NADPH	nicotinamide adenine dinucleotide phosphate
*Nrf1*	nuclear respiratory factor 1
OGTT	oral glucose tolerance test
PBMC	peripheral blood mononuclear cell
PGC1α	peroxisome proliferator-activated receptor gamma coactivator 1-alpha
PLS-DA	Partial Least Squares Discriminant Analysis
Q-TOF	quadrupole time-of-flight (mass spectrometer)
RONS	reactive oxygen and nitrogen species
ROS	reactive oxygen species
RYGB	Roux-en-Y gastric bypass
SCFA	short-chain fatty acids
SM	sphingomyelins
TCA	The citric acid cycle (CAC)/the tricarboxylic acid (TCA) cycle/Krebs cycle
TG	triglyceride
T2DM	type 2 diabetes mellitus
